# 899. Epidemiology of the 2022 Mpox Outbreak in the US Veterans Health Administration (VHA)

**DOI:** 10.1093/ofid/ofad500.944

**Published:** 2023-11-27

**Authors:** Cynthia A Lucero-Obusan, Gina Oda, Patricia Schirmer, Connor W Edson, Christina Trevino, Mark Holodniy

**Affiliations:** U.S. Department of Veteran Affairs, Public Health National Program Office, Palo Alto, California; Department of Veterans Affairs, Palo Alto, CA; Department of Veterans Affairs, Palo Alto, CA; Department of Veterans Affairs, Palo Alto, CA; Veterans Affairs, Palo Alto, California; Department of Veterans Affairs, Palo Alto, CA

## Abstract

**Background:**

Mpox, an orthopoxvirus zoonotic disease, is endemic to Africa. In May 2022, cases were reported concurrently in endemic and non-endemic countries, including the US. WHO and DHHS subsequently declared Mpox a public health emergency. Herein, we examine Mpox in the Veterans Health Administration (VHA).

**Methods:**

Mpox diagnostic and whole genome sequencing (WGS) results, demographics, hospitalizations, deaths, diagnosis codes, exposure information, pharmacy and immunization data were obtained from VHA data sources and/or chart review (5/23/22-4/11/23). Association of risk factors with Mpox was determined using multivariable logistic regression.

**Results:**

Of 1,034 Veterans tested, 251 (24%) were orthopox positive in 32 states (peaking in August 2022), with 50.2% HIV+ and 32.7% with other concurrent sexually transmitted infections (STIs). Significant associated Mpox risk factors included: male gender, age < 55 years, non-Hispanic Black race/ethnicity, and HIV positivity. Among 193 with confirmed Mpox, 90% reported intimate contact and/or an epi link to another Mpox case; 83% were men who have sex with men (MSM); 24% received Tecovirimat; and 11% were hospitalized with no deaths attributable to Mpox. 13% had reported or documented smallpox vaccine(s) >2 weeks prior to exposure (primarily as active duty) and an additional 14 (7%) received JYNNEOS < 2 weeks prior to presentation (or precise date not recorded). Review of 80 patients with evaluable WGS results found all were clade IIb, representing 10 different lineages from 18 states and District of Columbia. Serial WGS samples (n=3) evaluated after Tecovirimat regimens revealed no drug resistance. Since July 2022, over 4,300 Veterans have received at least 1 dose of smallpox/Mpox vaccine with over 6,700 doses having been administered at a VA facility.

**Figure:**
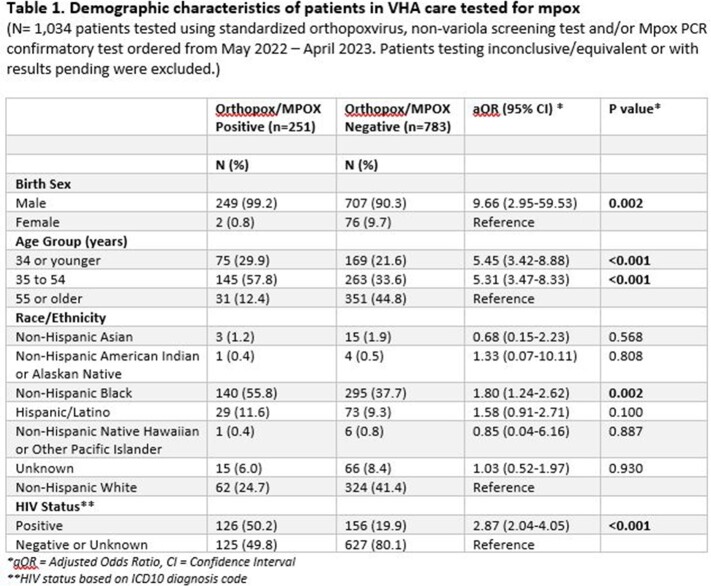

**Figure:**
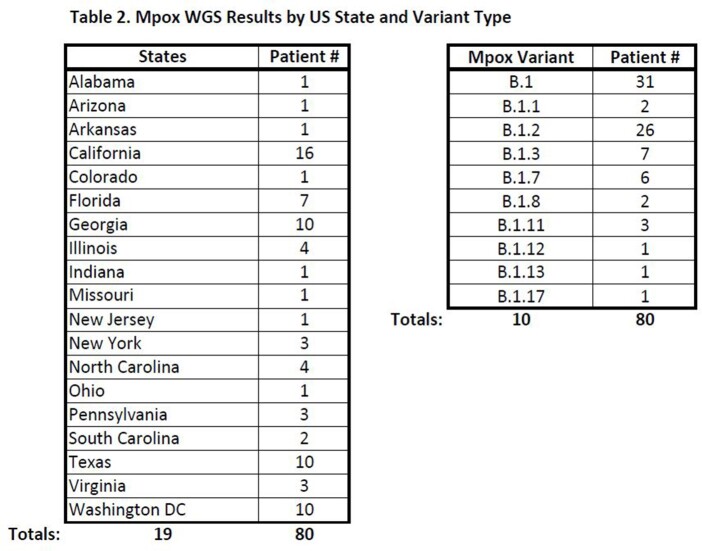

**Conclusion:**

The Mpox outbreak primarily affected younger, MSM, non-Hispanic Black race and HIV+ males among those in VHA care. Although some were vaccinated, the protective effect may have been diminished, or it was received too close to the exposure to provide adequate protection. Viral diversity was noted across geographic regions. Veterans with risk factors would benefit from vaccination and risk reduction strategies for Mpox and other STIs.

**Disclosures:**

**All Authors**: No reported disclosures

